# An International Medical Degree

**Published:** 1920-10-16

**Authors:** 


### The Hospital

The Workers' Newspaper of Administrative Medicine and Institutional Life, Administration, National Insurance and Health.

No. 1793, Vol. LXIX. SATURDAY, OCTOBER 16, 1920. PRICE TWOPENCE

### AN INTERNATIONAL MEDICAL DEGREE.

Medicine is among the most universal of professions, and the benefits it can confer on mankind are becoming daily and obviously more numerous. During the past thirty years its forces have overcome some of the most terrible enemies of mankind, and all will admit that these results have been achieved by workers, tireless in their efforts, sparing neither themselves nor their substance.

But throughout all this work, although the energy has been boundless and the enthusiasm the keenest, we have had repeated examples of physicians in different countries working with one and the same object in view, and yet quite ignorant of their partnership in the cause of science. Recently it has been repeatedly emphasised, by those well qualified to speak, that medical research can be intelligently prosecuted by all grades of the profession. Clinical, or bed-side, research, is a type quite as important as laboratory research, although at present, unfortunately, one finds laboratory research greatly in excess. Without doubt this situation will be remedied, but even then, in order to achieve the greatest benefit to mankind, it should be possible to transmit the result of all research to an International Board, who could justly investigate and correlate the results obtained.

It is a suggestion worthy of consideration that this work and the spirit of reciprocity therein could be developed by the establishment of an international medical diploma. We see in a diploma of this type a very great assistance to young qualified men, of whom many might thereby pursue a far truer international study of their profession, and also an improvement in scientific relations with our neighbours. The first step in this movement of reciprocity has already been made by the establishment of the Fellowship of Medicine, an institution which has sown the seed for a concerted movement which was needed long ago.

Also a diploma from an International Board would tend to provide a recognised status for all members of the profession who had achieved a sufficient distinction in their own country. An International Medical Council could issue a qualifying diploma or countersign, and thereby render universally valid all degrees or diplomas issued by the medical centres in different parts of the world, thus enabling the holder to practise unhampered the wider phases of investigational work, wherever and with whomsoever he worked. We note, therefore, with satisfaction a recent report dealing with the inspection and examination of medical schools and their methods in another country. In this way only can a nation bring its medical service up to the highest possible standard. That this standard must and can be a high one and a progressive and remarkably efficient one we have seen during the war. We have learnt the value in co-ordination of effort, and the lesson we would point out is the great good that would result from an improvedly reciprocal spirit among the medical services of the civilised peoples.

We have suggested the setting up of a Board of Examiners, consisting of members from Great Britain and her Dominions, from America, and from the European countries. This board might issue a qualifying diploma, either as the result of examination or after the inspection of diplomas or degrees already gained. This international diploma could enable its holder to practise in any country where the Board was recognised, and to move about and work among the different peoples who contributed to the formation of the Board. The advantages are obvious, and would be enormous. The work of the Rockefeller Institute in America, the Lister Institute in London, the Pasteur and other Continental institutes, together with the influence of all the great clinical teachers, would be more widely disseminated, and the resulting benefits should continually increase. Thus might we see a true and a greater co-ordination of effort, a spirit of reciproicty, an atmosphere of friendly enthusiasm, among the medical forces of all the civilised peoples, a result both foreshadowed and deserved after the diverse medical achievements of the last thirty brilliant years.

A further step in the movement of universal reciprocity in medicine would be made by establishing in the chief capitals of the world hospitals named and conducted by the countries contributing to the formation of the Board of Examiners. This movement is already being foreshadowed here by the proposed establishment of an American hospital in London. Such hospitals would give every practitioner the opportunity to work and to be seen at work in his own natural atmosphere and surroundings. Thus the medical profession might in time be welded into one great body working yet more efficiently for the benefit of mankind.

